# The impact of acute bike desk usage before encoding and during early consolidation on memory task performance in university students and use case evaluation in an educational setting

**DOI:** 10.1371/journal.pone.0319658

**Published:** 2025-03-17

**Authors:** Ahmed Mohsen Abbas El-Hagrasy, Rachel Anna Marshall, Thuraiya Hilal Said Al-Rawahi, Sally Doherty, Nitya Kumar, Declan Gaynor

**Affiliations:** School of Medicine, Royal College of Surgeons in Ireland Medical University of Bahrain, Busaiteen, Bahrain; Università degli Studi di Milano: Universita degli Studi di Milano, ITALY

## Abstract

This study examined the impact of using bike desks on cognitive function and memory among university students. Physical activity during adolescence offers enduring health benefits, yet sedentary behaviors prevail among young adults, posing significant health risks. Bike desks, integrating stationary cycling with ergonomic desk designs, aim to mitigate sedentary behavior while enhancing cognitive performance. Research indicates that acute aerobic exercise improves executive functions, memory, and attention, which is particularly beneficial in educational settings. The study employed the verbal paired associates learning task (VPAT) to assess memory performance when either bike desk usage at moderate intensity (intervention) or rest (control condition) occurred before encoding and during early consolidation in 26 young adult medicine and nursing students in a library setting. We hypothesised that bike desk usage will enhance memory encoding and consolidation compared to the control condition of rest. The results of our study showed no significant differences in VPAT scores or response latency between seated and bike desk conditions. Supplementary analysis, including a multiple linear regression model (*R*^*2*^: 0.773, Adjusted *R*^*2*^: 0.651, p <  0.001) revealed that higher BMI, more frequent bicycle or stationary bike usage, and higher physical activity category were associated with improved VPAT performance with the bike desk, while higher vigorous MET minutes per week negatively impacted performance. This analysis suggests there are potentially numerous uncharacterized modulators of the impact of exercise on memory, warranting further research to identify and understand these factors.

## Introduction

Physical activity during adolescence is widely recognized for its numerous health benefits, including improvements in cardiorespiratory fitness, muscular strength, bone health, heart function, weight management, cognitive development and promoting prosocial behavior, with many of these benefits persisting into adulthood [1–4].

Conversely, sedentary lifestyles have become a growing concern noted in around 31% of young adults. It contributes to approximately 3.2 million deaths yearly, accounting for 6% of global mortality [1,3]. Sedentary behavior, defined as sitting or reclining with an energy expenditure of 1.5 metabolic equivalent tasks (METs) or less [1], poses significant health risks, including obesity, cardiovascular diseases, diabetes mellitus, and certain cancers, thereby contributing to increased mortality rates [4].

University students, who often spend prolonged periods attending lectures and studying, are particularly prone to sedentary behaviors. Studies suggest that many students struggle to incorporate physical activity into their daily routines due to academic demands [2]. The World Health Organization recommends that adults engage in at least 150-300 minutes of moderate-intensity aerobic exercise, 75-150 minutes of vigorous-intensity aerobic exercise per week, or an equivalent combination, alongside minimizing sedentary behavior. [5]. Even light-intensity physical activity can offer health benefits when replacing inactive periods [6]. To mitigate the negative health impacts of prolonged sedentary behavior, adults are encouraged to exceed these recommended levels of physical activity.

Research literature has consistently demonstrated that physical exercise enhances cognitive function and overall well-being. Regular aerobic exercise promotes neuroplasticity, mitigates the effects of normal and pathological ageing, improves cognitive abilities, and enhances mood [7]. Aerobic exercise has also been shown to significantly improve processing speed, crystallised intelligence, attention, executive function including problem solving and response inhibition, with benefits increasing with age [8–10].

Research has also explored the effects of acute exercise on memory, with studies showing that it predominantly enhances both short-term and long-term declarative memory [9,11–15]. Declarative memory involves the recollection of experiences (episodic) and facts (semantic) and consists of phases conceptualised as encoding, consolidation, and retrieval. During encoding, sensory information is processed by the hippocampus and surrounding medial temporal lobe structures, which play a critical role in transforming experiences into memory traces. Acute exercise before encoding is proposed to affect encoding through enhanced attention, increased production and release of neurotransmitters and proteins such as brain-derived neurotrophic factor (BDNF) in addition to activation of receptors such as N-Methyl-D-aspartate (NMDA) [16,17]. Consolidation is primarily supported by the hippocampus and interactions with the neocortex. Early-stage consolidation strengthens memory traces at a synaptic level in the minutes to hours following encoding through the process of long-term potentiation and modulated by BDNF and NMDA [16]. During late-stage or systems consolidation (hours to days following encoding), memory traces are gradually reorganised and transferred from the hippocampus to distributed networks in the neocortex, making them available for retrieval over time [18]. Acute exercise performed shortly after encoding is proposed to stabilise new memory traces through increased production of neurotrophins and growth factors, including, BDNF, phosphorylated cAMP response element-binding (CREB) and post-synaptic protein 95 (PSD-95) [19,20].

Several modulators of the effect of exercise on memory task performance have been identified, including temporality, duration, mode, and intensity. Evidence suggests that enhancements in memory are observed when exercise is undertaken before memory encoding and during consolidation [14]. Shorter bouts of exercise ( < 20 minutes) were identified to provide greater enhancements in memory compared to bouts of longer duration ( > 40 minutes) [13] while there is some evidence to suggest that high-intensity exercise may improve episodic memory, and moderate-intensity exercise may improve working memory capacity [15]. Finally, different modes of exercise were shown to have variable effects on memory, with cycling being the most effective at improving long-term memory while walking was associated with more significant improvements in short-term memory [13].

The rising popularity of active workstations, including bike desks, reflects their dual benefits of reducing sedentary behavior and the potential for enhancing cognitive function through physical activity. Research suggests that integrating active workstations into office settings decreases sedentary time and health risks associated with prolonged sitting without negatively impacting cognitive performance in the areas of memory, executive function, reading comprehension and mathematics [21–23]. While standing desks, in general, did not negatively impact productivity measures, cycling and walking workstations have predominantly resulted in reductions in typing and mouse-based computer tasks [21].

Compared to the workplace, the use and assessment of bike desks in educational settings is quite limited. A small number of studies have shown that implementing bike desks in school and university classrooms can positively impact students’ physical activity without compromising their academic performance or engagement in class [24,25]. A limited number of studies reported on implementing bike desks in library settings. Generally, these studies investigated students’ perceptions of the bike desks and revealed that bike desks were positively received by students who reported they were more likely to visit the library due to their presence [26–28]. One study reported that while students perceived using a bike desk to be less effective for studying than a traditional seated desk, their use did not impact academic performance [29]. The paucity of research studies assessing the impact of active workstations, including bike desks, in educational environments is an area which requires attention, considering the potential benefits their application may have in addressing sedentary behaviours prevalent in young adults in tertiary education.

Our study’s primary aim was to investigate the effects of acute bouts of exercise performed on a bike desk at light to moderate intensity before memory encoding and during early-stage consolidation on episodic memory task performance in a library setting. Scheduling the exercise before encoding and during consolidation was designed to simulate periodic changes between physical activity and sedentary behaviour that may occur during a study session while using a bike desk and seated desk in a library setting. Light to moderate exercise intensity was chosen as it is more conducive to an academic library environment, where excessive physical exertion could be disruptive and counterproductive to the primary purpose of studying and learning. We hypothesized that using a bike desk will positively influence long-term episodic memory compared to being seated. The study also aimed to report students’ evaluation of engaging in learning activities while using a bike desk in a library setting.

## Materials and methods

### Participants

The effect sizes for the impact of exercise on memory task performance vary greatly within the literature, with a number of different moderators and temporal effects observed. A similar study to our research study reported Cohen’s d =  1.04 for cycling before memory encoding, and 0.24 after encoding compared to control conditions [30]. When assuming an effect size Cohen’s d =  0.8, significance =  0.05 and power =  0.8 a value of minimum sample size of 25 per treatment group was calculated as outlined in supporting information, S1 File.

All aspects of the research study, including recruitment, consent, experimental protocols and data management, received ethical approval from the RCSI Bahrain research ethics committee (REC/2023/180/07-Jun-2023) in June 2023. The study was conducted according to the principles expressed in the Declaration of Helsinki.

Self-selection sampling was used to recruit a pool of willing participants for the study via the university email network. All participants were provided with a participant information leaflet and an electronic written consent form. Twenty-six students from the School of Medicine and the School of Nursing, 18 years of age and above, provided electronic written consent through the MS Forms platform prior to joining the study. Participants were awarded a shopping voucher after completion of the study to acknowledge their participation. Recruitment of participants started on the 24^th^ of September, 2023, and closed on the 25^th^ of September, 2023. The experimental phase of the study was conducted from the 7^th^ of October, 2023, to the 28^th^ of October, 2023.

### Equipment

A height-adjustable standing desk was combined with a stationary bike (Star Trac Spinner Pro + stationary bike). The height and position of the bike and desk were adjusted to ensure participants were in a comfortable and appropriate position for engaging in moderate-intensity cycling. A chest strap heart rate monitor (Polar H10 heart rate sensor, Polar Electro Oy, Kempele, Finland) was paired with the Polar Beat app [31] to allow recording of participants’ heart rate during experiments.

### Verbal paired associates learning task

The verbal paired associates learning task (VPAT) was licensed from Millisecond LLC and administered on a laptop computer using a local installation of Inquisit 6 [32]. Participants were presented with a list of 14-word pairs, one-word pair at a time, for a set display time and in a fixed sequence on the computer screen. Once the list had finished, the first word of each word pair was presented as a prompt together with a textbox, and participants were asked to type the missing second word. Feedback was provided during the immediate recall trials. This list and recall task were repeated three times before a break of 20 minutes. After the break, participants worked through a delayed recall test with no provided feedback and a recognition task in which 28 words were presented to them, one by one, and participants decided whether the word was part of the original list by pressing ‘Y’ for Yes and ‘N’ for No. Of the presented words, 14 were listed words and the other 14 were new distractor words. The test ended with a free recall test in which participants typed as many of the original list word pairs into a single textbox as possible.

The number of correct answers provided by each participant in all four sections (immediate recall, delayed recall, recognition, and free recall) of the VPAT were recorded along with latency data (time taken to complete responses) for each section of the task.

### Orientation

Participants initially attended an orientation session at the experiment venue in the university library to allow familiarization with the cycling desk, the experiment location and the VPAT. During orientation, participants also completed a survey related to demographics (age, height, weight) and the International Physical Activity Questionnaire, IPAQ, [33] to establish their level of physical activity.

Participants were fitted with a heart rate monitor before starting their orientation. Participants were instructed to cycle at approximately 80 rpm. After 5 minutes, the participants were asked to rate their perceived effort in RPE (rating of perceived exertion) on the Borg Scale, and their heart rate was recorded. The resistance of the stationary bike was increased manually by the research team, and after 1 minute, the participant was asked to provide a new RPE and their heart rate was recorded. This step was repeated until participants provided at least 5 RPE values and an RPE of 15 or more, after which the participants were requested to cycle until they had completed a total of 20 minutes of cycling with an RPE in the range 11-13.

### Experimental protocol

**Fig 1:**
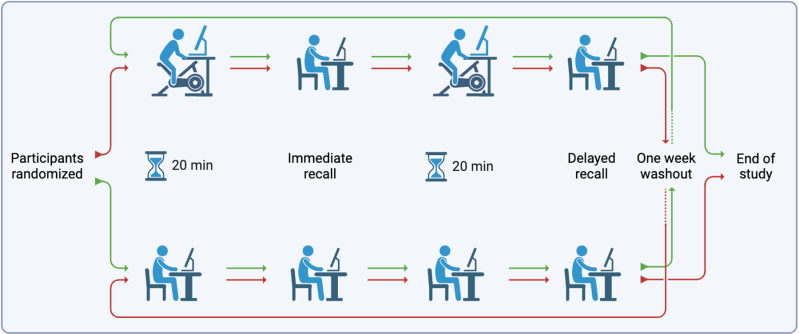
Cross-over randomized control design. Participants (n =  26) were randomized into one of two paths (red, n =  13; green, n =  13), completing both bike desk (intervention) and seated (control) conditions. (Created in BioRender. Bahrain team 1, R. (2024) BioRender.com/e72a479).

Following orientation, each participant visited the experiment location in the university library on two different occasions, separated by approximately one week to undertake the intervention and control conditions of the research study. The sequence of administration of intervention and control conditions was randomly assigned for each participant to achieve a counter-balanced randomized controlled protocol design, Fig 1.

The experiment protocols consisted of the following steps for both intervention and control conditions:

(1.) Participants watched self-selected anatomy or physiology videos from an online medical educational resource, and, if desired, made summary notes for 20 minutes while seated (control) or cycling at a bike desk at a moderate intensity, which they rated as 11-13 RPE on the Borg Scale (intervention). Video length and subject difficulty varied depending on the chosen topic, with participants watching one or more videos to fill up the total duration of 20 minutes, allowing for a variation in content and cognitive load across participants.
(2.) Participants completed the first section of the computer-based VPAT, duration ~ 10 minutes, while seated (control and intervention).
(3.) Participants continued watching anatomy or physiology videos and, if desired, made summary notes for 20 minutes while seated (control) or cycling at a bike desk at moderate intensity, which they rated as 11-13 RPE on the Borg Scale (intervention).
(4.) Participants completed the second section of the computer-based VPAT, duration ~ 10 minutes, while seated (control and intervention).

The participants heart rate was recorded using a chest strap heart rate monitor (Polar H10 heart rate sensor, Polar Electro Oy, Kempele, Finland) paired with the Polar Beat app [31], and participants perceived exertion was recorded every 5 minutes on the Borg scale.

### Bike desk evaluation

After completion of the orientation, and both seated and bike desk experiments, participants completed a short questionnaire designed to report their perceptions of the bike desk, its suitability, any impact on their attention or performance in the memory tests and prospective usage of bike desks in different settings in the future. All items recorded in the evaluation survey are available in supporting information, S2 Apprendix.

### Statistical analysis

Statistical analyses were completed using IBM Statistics SPSS 29 [34]. The Kolmogorov -Smirnov test was used to test normality of the heart rate, VPAT scores, latency, and change in VPAT scores and response latency between conditions. Heart rate and VPAT scores are presented as median and interquartile ranges (IQR), and latency are presented as mean ± SD, with statistical significance set to 0.05.

Participants’ measured heart rate, scores in each section of the VPAT and latency in the recognition section of the VPAT during seated and bike desk conditions were compared using the related-samples Wilcoxon signed rank test due to non-normal distribution of these data. Participants’ latency for all sections of the VPAT during seated and bike desk conditions were normally distributed, so the paired-samples t-test was used to investigate differences between the conditions.

#### Supplementary analysis.

To further explore any relationship between participant characteristics and changes in performance and latency in the VPAT between seated and bike desk conditions, a post hoc multiple linear regression was conducted. Model fit was reported as adjusted R-square, and the statistical significance of the predictors was assessed using a threshold of p <  0.05. The standardized coefficients (β) of predictors were reported to indicate their relative effect size or contribution to the models.

## Results

### Participant characteristics

**Fig 2:**
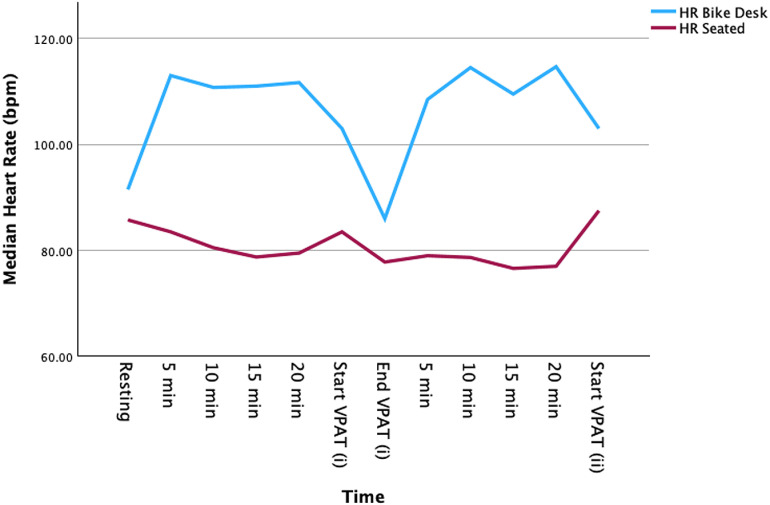
Median heart rate during seated and bike desk conditions.

All participants (12 male and 14 female) who attended orientation completed the study. Participants’ age ranged from 18 – 37 years and had a mean age =  21.5 ±  4.0 yr. The mean BMI of participants was 25.3 ±  5.6 kgm^-2^ with two participants being underweight, eleven normal weight, nine pre-obese and four obese. According to the IPAQ guidelines, of the 24 valid participant IPAQ responses, four had low, thirteen had moderate, and seven had high levels of physical activity (PA). Four participants reported never using a bicycle or stationary bike, eight reported rarely, another eight reported occasionally, and three reported often. The median heart rate of participants during the two 20-minute periods of cycling in the bike desk condition was significantly higher than those periods during the seated condition, 111 (103 – 127) bpm vs 79 (73 – 88) bpm, (W =  21763, *z* =  12.506, p <  0.001), Fig 2.

### Memory task performance

**Fig 3:**
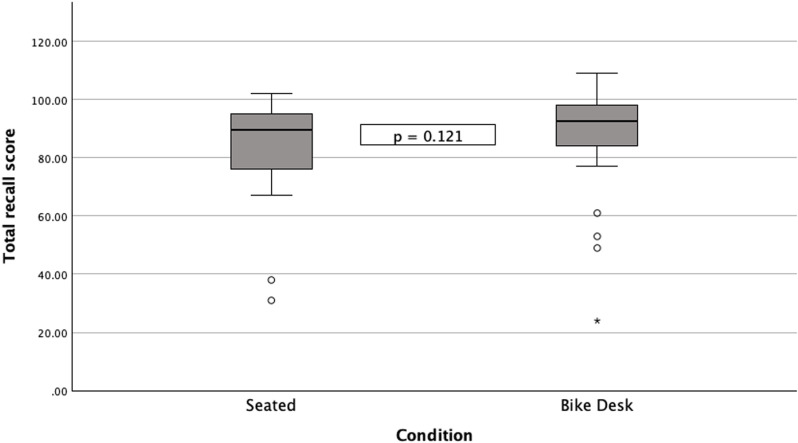
Comparison of VPAT total recall scores.

Median scores and interquartile ranges for all sections of the VPAT and the total test score are reported for seated and bike desk conditions, Table 1. Related-samples Wilcoxon signed rank tests showed no significant differences between seated and bike desk conditions in total VPAT scores (W =  236.5, *z* =  1.550, p =  0.121), Fig 3, and across individual section scores.,

**Table 1:** Summary of VPAT scores and response latency.

	VPAT Score				VPAT response latency (ms)	
	Mean ± SD		Median (IQR)		Mean ± SD	
	Seated	Bike desk	Seated	Bike desk	Seated	Bike desk
**Immediate recall**	36 ± 11	39 ± 13	37 (33 – 43)	42 (36 – 47)	205,577 ± 51,603	206,772 ± 79,904
**Delayed recall**	12 ± 3	12 ± 4	13 (11 – 14)	13 (11 – 14)	49,264 ± 15,045	44,858 ± 15,772
**Recognition**	27 ± 2	28 ± 2	28 (28 – 28)	28 (28 – 28)	16,618 ± 14,519	14,570 ± 5,357
**Free recall**	9 ± 3	9 ± 3	9 (8 – 11)	9 (7 – 11)	101,710 ± 57,741	88,621 ± 37,024
**Total recall**	84 ± 17	87 ± 20	90 (76 – 95)	93 (84 – 98)	373,168 ± 76,855	354,821 ± 103,154

### Memory task response latency

**Fig 4:**
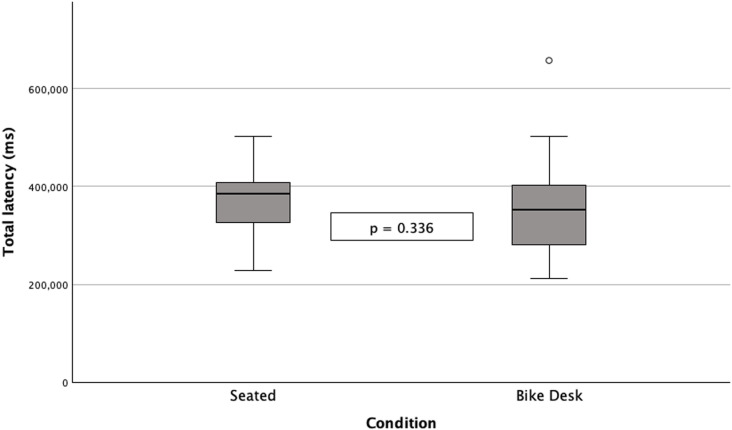
Comparison of total latency (milliseconds) in VPAT during seated and bike desk conditions.

No significant differences were observed between seated and bike desk conditions in response latency in the VPAT as a whole or in any section of the VPAT as determined using paired-samples t-test (total recall, immediate recall, delayed recall, and free recall) and related-samples Wilcoxon signed rank test (recognition). While higher latency was observed for the seated condition in all sections, with the exception of immediate recall, the differences observed were not significant, Fig 4.

### Supplementary analysis: Modulators of memory task performance

A multiple linear regression analysis was conducted to explore the relationship between participant characteristics and differences in VPAT performance during the seated and bike desk conditions. The independent variables included in the model were BMI, IPAQ PA category, frequency of bicycle or stationary bike use, experiment order, heart rate difference between conditions (HR Bike Desk - HR Seated), vigorous MET minutes per week, moderate MET minutes per week, and walking MET minutes per week.

The overall model was statistically significant (F (8,15) =  6.371, p =  0.001), indicating that the predictor variables collectively explain a significant portion of the variance in difference in VPAT performance between conditions. The model accounted for approximately 77.3% of the variance, with an adjusted *R*^2^ =  0.651, Table 2.

**Table 2:** Factors associated with changes in total recall in VPAT between seated and bike desk conditions.

Independent variable	B	Std Error	*β*	t	p
(Constant)	-55.859	15.194		-3.676	.002
BMI	1.448	.323	.644	4.489	<.001
IPAQ PA category	14.577	4.969	.772	2.933	.010
Frequency of bicycle or stationary bike usage	4.402	1.906	.354	2.310	.036
Experiment Order	4.119	4.513	.164	.913	.376
HR bike desk - HR seated	-.141	.116	-.161	-1.217	.243
Vigorous MET minutes/week	-.015	.003	-1.126	-4.437	<.001
Moderate MET-minutes/week	-.006	.004	-.267	-1.532	.146
Walking MET-minutes/week	-.003	.003	-.154	-1.010	.328

*R*^*2*^: 0.773, Adjusted *R*^*2*^: 0.651, p <  0.001

Among the predictors, BMI (β =  0.644, p <  0.001) and IPAQ physical activity (PA) category (β =  0.772, p =  0.010) were significant positive predictors of differences in VPAT performance. This suggests that higher BMI and higher levels of physical activity, as categorized by IPAQ, are associated with greater increases in VPAT performance during the bike desk condition. The frequency of bicycle or stationary bike use also showed a positive and significant association with changes in total recall (β =  0.354, p =  0.036).

Conversely, vigorous MET minutes per week was a significant negative predictor (β =  − 1.126, p <  0.001), indicating that higher levels of vigorous physical activity were associated with decreases in VPAT performance in the bike desk condition. Other variables, including experiment order, HR Bike Desk - HR Seated, moderate MET minutes per week, and walking MET minutes per week, did not show significant associations with differences in VPAT performance (p >  0.05).

A similar multiple linear regression analysis consisting of the same predictors assessing their associations with VPAT response latency differences between seated and bike desk conditions was not found to be statistically significant (F (8,15) =  2.055, p =  0.109).

### Bike desk evaluation

Summary statistics of the evaluation questionnaire are presented in supporting information S2 Appendix. Participants’ feedback regarding bike desk usage was generally positive, with the exception of the seat, which was deemed to be uncomfortable by 18 (69.2%) of the participants. A total of 11 participants indicated that the bike desk did not distract them from focusing on task, while an equivalent number reported that it did distract them. Despite this, more than 75% of the participants did feel that they could engage in academic tasks while using the bike desk at ‘fairly light’ to ‘somewhat hard’ levels of perceived exertion. Of the participants, 13 (50%) reported they would use a bike desk in the library while engaging in academic tasks and 17 (65.4%) reported they would use a bike desk while taking a break from academic tasks. Participants indicated a strong preference for using a bike desk in a private space in the library (80.7%) or an active study gym in the sports hub (53.8%) as opposed to in an open area in the library (15.4%). Participants also showed a strong preference for engaging with educational content in the form of video (76.9%) and audio (65.4%) rather than reading (34.6%) and making notes (23.1%) while using a bike desk.

Participants’ perceptions of the impact of bike desk use during the experiments showed just under half (46.2%) felt they were more focused on the videos during the cycling experiment, and just over half (53.8%) felt they performed better in the memory test during the cycling experiment, compared to the seated experiment. Finally, 18 participants (69.2%) felt a bike desk would improve their study experience, and 20 participants, representing more than three-quarters of the study, reported that they would use it once a week or more.

## Discussion

### Study design

The design of this study aimed to simulate the real environment that students experience while studying by conducting the experimental sessions in a university library setting like a previous study by Pilcher et al., who investigated the effects of bike desk usage compared to a traditional seated desk on academic performance, meta-cognition, and sleep in a university library setting [29]. A randomized counter-balanced design was adopted to minimize the influence of order and carryover effects, such as fatigue or learned effects that may influence participants’ performance and perceptions when undertaking the randomly assigned bike desk cycling experiment or seated control first. This design differed from a similar study by Labban et al. [30] which involved three separate groups, an ‘exercise-prior to test’ group, an ‘exercise-after test’ group, and a control group. Comparatively, our counterbalanced design allowed participants to undergo both the experimental and control experiments while using a small sample size. Randomisation of the sequence of conditions helped distribute any carryover or practice effects evenly across conditions. Roughly one week was incorporated between conditions to reduce the likelihood of immediate carryover effects influencing subsequent experiments. To further address practice effects, we provided a standardized practice session before the study commenced, ensuring that participants were familiarised with the memory task before starting the experimental trials.

Moderate-intensity exercise, defined as an exercise intensity corresponding to 3 to 5.9 (less than 6) metabolic equivalents (METs), where one MET corresponds to an energy consumption of 1 kcal/kg per hour while an adult is resting, was selected for the 20-minute bike desk cycling sessions [35]. This level of intensity was chosen as it is more suitable for a library setting, and it has been suggested to be the most beneficial for working memory by comparison to high-intensity exercise [15].

Our experiment design was also influenced by literature indicating that exercise before memory encoding and during consolidation enhances memory function in young adults, whereas exercise during encoding negatively affects memory regardless of age [11]. The selected delay of 20 minutes between encoding and assessing long-term memory in our study was shorter than that in many studies but was generally in line with moderate-intensity exercise having beneficial effects on shorter delays between exposure and memory assessment [14].

Both heart rate and perceived exertion were measured to ensure participants achieved the moderate-intensity exercise and negative impacts resulting from fatigue on performance were avoided [36]. Ratings of perceived exertion (RPE) of 13-15 are considered moderate-intensity, however, an RPE level of 11-13, slightly below the moderate-intensity level of exercise was specifically targeted, as this level was deemed appropriate for our participants to feasibly replicate within the constraints of a library setting [37].

The VPAT [32] was selected for this study due to its widely recognized efficacy in assessing both encoding and early consolidation memory processes, as demonstrated in prior research studies [38–41]. The computerized format of the VPAT facilitated the collection of critical quantitative data, including response accuracy and latency, offering further insights into the effects of bike desk usage on memory performance.

### Memory task performance

Overall, our findings revealed no significant differences in VPAT performance between the seated and bike desk conditions across all sections. These results are inconsistent with meta-analysis, which suggests that young adults showed memory performance benefits when exercise is performed either before encoding and/or during consolidation. While in the minority, there are similar studies that have reported moderate-intensity exercise having no impact on memory task performance in young adults [39,42]. Although our study did not reveal significant overall effects, it is possible that the lower cognitive load of the VPAT memory task might have been insufficient to demonstrate such benefits as research on adolescents with ADHD indicates that cycling on a bike desk can benefit higher cognitive load phonological working memory tasks [43]. Another element of the experimental design that should be considered is the nature of the cognitive task (watching anatomy or physiology videos), which was undertaken during the seated or cycling period during early consolidation. Cognitive tasks performed during consolidation has been shown to impair memory performance compared to an offline-state waking rest [44]. This effect is attributed to the reactivation of recently formed memories without interference from new sensory input or cognitive load in the offline state. The educational videos resource used during consolidation was selected due to its relevance to the participants’ programme of study, but the difficulty and cognitive load of the selected videos were not controlled and may have introduced varying degrees of interference during consolidation and could potentially have negated any potential benefits provided by the moderate intensity exercise in the bike desk group.

Interestingly, the strong correlations between seated and bike desk conditions across all VPAT sections suggest that memory performance remains stable regardless of whether a person is seated or engaging in moderate-intensity cycling. This stability supports the view that the presence of a bike desk does not adversely alter cognitive performance on memory tasks.

The lack of improvement in memory encoding and consolidation with the bike desk condition might be related to the intensity of the exercise. The average heart rate in our cycling condition was 111 (IQR 103 – 127) bpm, similar to other studies reporting moderate-intensity exercise on a bike desk with heart rates of 116.8 ±  16.0 and 116 ±  2.9 bpm, respectively [45,46]. While the prescribed bike desk experimental conditions provided a moderate level of physical activity, it may not be vigorous enough to significantly impact memory encoding and consolidation. Loprinzi et al. highlighted that acute vigorous-intensity exercise can enhance episodic memory during the early consolidation period, suggesting that more intense physical activity might be necessary for substantial cognitive benefits [11]. A recent field study conducted on endurance runners in their natural training environment reported superior memory performance following acute high-intensity exercise compared to moderate-intensity exercise [47]. This evidence is supported by a recent meta-analysis which reported that acute high-intensity exercise is more effective than moderate-intensity continuous exercise at increasing BNDF, a neurotrophin that is intimately involved in memory processes [48]. Conversely, another review found that acute low-intensity exercise had a more pronounced effect on short-term memory, reflecting that the type and timing of exercise can influence memory differently [13]. This dichotomy in findings underscores the complexity of how exercise intensity and duration interact with memory processes.

Latency values in the bike desk condition, which were slightly lower, though not statistically significant, are consistent with previous literature showing decreased reaction times on cognitive tests for those using a bike desk compared to a traditional seated desk [45]. Another study involving athletes also saw faster responses on the working memory tasks [49]. This phenomenon could be explained by the increased arousal and mental engagement associated with light physical activity, which may facilitate quicker cognitive responses [50]. Further research is needed to explore the relationship between exercise and cognitive processing speed.

### Suppementary analysis; modulators of memory task performance

We also explored potential modulators of memory task performance between seated and bike desk conditions. The literature has identified age as a modulator of memory function when examining the impact of exercise on memory. Young adults demonstrated improvements in episodic memory when exercise occurs before encoding or during consolidation, while older adults experienced impairments in episodic memory [11,13]. Gender has not been identified as a modulator in the literature, and our analysis also finds no association between gender and changes in memory performance [11]. Habitual physical activity has not been examined in the literature as a modulator of the impact of exercise on memory function. Subjective and objective measures of physical activity have been assessed as potential modulators of memory performance in studies on young adults, but no association between free-living physical activity and memory task performance or self-reported memory problems were reported [39,51]. Our observation that higher self-reported physical activity, was associated with greater increases in memory performance on the bike desk is an interesting observation that may be linked to another modulator, cardiorespiratory fitness. A review reported an association between cardiorespiratory fitness and memory performance but also noted that none of the studies controlled for habitual physical activity levels [52], suggesting a link between the two. The fitness level of participants has been assessed as a modulator of memory performance in temporal proximity to exercise in a meta-analysis, with average levels of fitness being associated with greater benefits to memory performance compared to higher levels of fitness. However, this association is tenuous as only a few studies were included in this analysis, and none of the studies reported a direct comparison between different fitness levels in the same experiment. A recent study examined the impact of different exercise intensities on cognitive function, including working memory, across low and high cardiorespiratory fitness groups [53]. No modulating effects were found for high and low-fitness groups in memory accuracy after exercise of different intensities. However, the low-fitness group experienced greater reductions in response time compared to the high-fitness group at moderate-intensity exercise, while this trend was reversed for high-intensity exercise [53]. The evidence from this recent study may also be related to our observation that higher levels of vigorous MET min per week were associated with reduced improvements in memory performance in the bike desk condition, which also raises intriguing questions related to the interplay of exercise intensity, cardiorespiratory fitness and potential memory performance benefits from using a bike desk.

The association we observed between higher BMI and greater improvements in memory performance while using the bike desk presents a counterintuitive finding that can be explored from multiple angles. One possible explanation is that individuals with higher BMI may experience greater cognitive and metabolic benefits from the added physical activity provided by the bike desk. This increased benefit could be due to the greater cognitive stimulation and metabolic changes induced by the exercise. Research on children has shown that a higher BMI can be linked to decreased cortical thickness, particularly in the prefrontal cortex, which is vital for executive functions such as working memory [54]. This finding suggests that a higher BMI might negatively impact cognitive abilities related to executive function in children. Conversely, in older adults (aged 61-84), a higher BMI has been positively associated with better performance in both verbal and non-verbal learning and memory tasks. This phenomenon is consistent with the “obesity paradox,” which posits that a higher BMI may offer some protective effects against cognitive decline in older age [55]. Furthermore, moderate physical activity, such as cycling, is known to enhance cognitive function by increasing cerebral blood flow and stimulating the release of neurotrophic factors that support brain plasticity. This effect might be particularly pronounced in individuals who are less physically active or have a higher BMI [56]. A review focused on overweight adolescents also found that physical activity improved core executive functions, including working memory, suggesting that physical activity can mitigate some of the cognitive drawbacks associated with higher adiposity [57]. These findings underscore the complex relationship between BMI, physical activity, and cognitive function and suggest that individual differences in baseline activity levels and physical fitness can significantly influence the cognitive benefits derived from exercise interventions like the use of a bike desk.

### Bike desk evaluation and future implementation

Research shows that incorporating physical activity into study routines can enhance cognitive function without negatively impacting memory [45] or academic performance [29]. Our findings align with this evidence, demonstrating that bike desks do not hinder memory performance and were generally well accepted by the student participants of the study with more than half indicating that they could engage in academic tasks while using the bike desk. This is similar to that reported by Pilcher et al. in their 10-week study during which no differences in motivation, focus or perceptions of preparedness for examinations were identified between the bike desk and traditional desk groups. Therefore, we believe bike desks can be an effective tool for integrating physical activity into academic and non-academic settings, while also having the potential to enhance cognitive function and productivity. For institutions, implementing bike desks in libraries could significantly improve student health without compromising academic performance by reducing sedentary behavior, alleviating stress, and improving well-being.

### Study limitations

The small sample size, consisting of 26 students, and the demographic of our participants, aged 18 and above from the School of Medicine and the School of Nursing, may restrict the generalizability of our results. This study was focused on acute bike desk usage and memory task performance and did not examine the potential impact that long-term or habitual use of a bike desk may have on cognitive function in an academic setting. This study was designed to simulate bike desk usage in a library setting and while we attempted to control for environmental distractions by conducting experiments in a restricted area within the library, we do acknowledge the potential for participants to have experienced variable external noise during the experiments. External factors such as sleep patterns, diet, and caffeine intake [58–60], which have been proven to affect performance in cognitive function tests were not controlled for. Finally, the observation that the level of habitual physical activity was associated with memory task performance changes between conditions should include the caveat that the physical activity measures were self-reported and subject to potential bias such as recall and social desirability.

## Conclusions

Our results do not provide evidence that acute bike desk usage at moderate intensity before encoding and during early consolidation either positively or negatively impact memory. However, post hoc analysis of modulators, identified possible associations between a number of participant characteristics (BMI and self-reported physical activity) that have not been extensively reported in the literature. Future research is needed to learn more about participant characteristics (cardiorespiratory fitness, metabolic health, objectively measured physical activity) that may act as modulators of the impact of bike desk usage on cognitive function. The potential impact of long-term bike desk usage at varying intensities and durations on students’ health and wellness, cognitive function and academic performance in educational settings also requires further attention.

## Supporting information

S1 File Study sample size calculation. (DOCX)

S2 Appendix Table 1 Summary data of participants’ responses to evaluation questionnaire part 1; Table 2 Summary data of participants’ responses to evaluation questionnaire part 2. (DOCX)

## References

[1] Isath A, Koziol KJ, Martinez MW, Garber CE, Martinez MN, Emery MS, et al. Exercise and cardiovascular health: A state-of-the-art review. Prog Cardiovasc Dis. 2023;79:44–52. doi: 10.1016/j.pcad.2023.04.008 37120119

[2] Okely AD, Ghersi D, Loughran SP, Cliff DP, Shilton T, Jones RA, et al. A collaborative approach to adopting/adapting guidelines. The Australian 24-hour movement guidelines for children (5-12 years) and young people (13-17 years): An integration of physical activity, sedentary behaviour, and sleep. Int J Behav Nutr Phys Act. 2022;19(1):2. doi: 10.1186/s12966-021-01236-2 34991606 PMC8734238

[3] Committee PAA. 2018 Physical Activity Guidelines Advisory Committee Scientific Report. Washington DC: US Department of Health and Human Services, 2018.

[4] WHO guidelines on physical activity and sedentary behaviour. Geneva: World Health Organization, 2020.

[5] Bull FC, Al-Ansari SS, Biddle S, Borodulin K, Buman MP, Cardon G, et al. World Health Organization 2020 guidelines on physical activity and sedentary behaviour. Br J Sports Med. 2020;54(24):1451–62. doi: 10.1136/bjsports-2020-102955 33239350 PMC7719906

[6] Ekelund UA-O, Tarp J, Steene-Johannessen J, Hansen BH, Jefferis B, Fagerland MW, et al. Dose-response associations between accelerometry measured physical activity and sedentary time and all cause mortality: systematic review and harmonised meta-analysis. British Medical Journal. 2019;(1756-1833 (Electronic)). 10.1136/bmj.l4570 PMC6699591 31434697

[7] Mandolesi L, Polverino A, Montuori S, Foti F, Ferraioli G, Sorrentino P, et al. Effects of Physical Exercise on Cognitive Functioning and Wellbeing: Biological and Psychological Benefits. Front Psychol. 2018;9:509. doi: 10.3389/fpsyg.2018.00509 29755380 PMC5934999

[8] Stern Y, MacKay-Brandt A, Lee S, McKinley P, McIntyre K, Razlighi Q, et al. Effect of aerobic exercise on cognition in younger adults: A randomized clinical trial. Neurology. 2019;92(9):e905–16. doi: 10.1212/WNL.0000000000007003 30700591 PMC6404470

[9] Chang YK, Labban JD, Gapin JI, Etnier JL. The effects of acute exercise on cognitive performance: a meta-analysis. Brain Res. 2012;1453:87–101. doi: 10.1016/j.brainres.2012.02.068 22480735

[10] Smith PJ, Blumenthal JA, Hoffman BM, Cooper H, Strauman TA, Welsh-Bohmer K, et al. Aerobic exercise and neurocognitive performance: a meta-analytic review of randomized controlled trials. Psychosom Med. 2010;72(3):239–52. doi: 10.1097/PSY.0b013e3181d14633 20223924 PMC2897704

[11] Loprinzi PD, Blough J, Crawford L, Ryu S, Zou L, Li H. The Temporal Effects of Acute Exercise on Episodic Memory Function: Systematic Review with Meta-Analysis. Brain Sci. 2019;9(4):87. doi: 10.3390/brainsci9040087 31003491 PMC6523402

[12] Loprinzi PD, Loenneke JP, Storm BC. Effects of acute aerobic and resistance exercise on episodic memory function. Q J Exp Psychol (Hove). 2021;74(7):1264–83. doi: 10.1177/1747021821994576 33535923

[13] Roig M, Nordbrandt S, Geertsen SS, Nielsen JB. The effects of cardiovascular exercise on human memory: a review with meta-analysis. Neurosci Biobehav Rev. 2013;37(8):1645–66. doi: 10.1016/j.neubiorev.2013.06.012 23806438

[14] Roig M, Thomas R, Mang CS, Snow NJ, Ostadan F, Boyd LA, et al. Time-Dependent Effects of Cardiovascular Exercise on Memory. Exerc Sport Sci Rev. 2016;44(2):81–8. doi: 10.1249/JES.0000000000000078 26872291

[15] Loprinzi PD. Intensity-specific effects of acute exercise on human memory function: considerations for the timing of exercise and the type of memory. Health Promot Perspect. 2018;8(4):255–62. doi: 10.15171/hpp.2018.36 30479978 PMC6249493

[16] Loprinzi PD, Frith E. A brief primer on the mediational role of BDNF in the exercise-memory link. Clin Physiol Funct Imaging. 2019;39(1):9–14. doi: 10.1111/cpf.12522 29719116

[17] Cunha C, Brambilla R, Thomas KL. A simple role for BDNF in learning and memory? Front Mol Neurosci. 2010;3:1. doi: 10.3389/neuro.02.001.2010 20162032 PMC2821174

[18] Reyes-Resina I, Samer S, Kreutz MR, Oelschlegel AM. Molecular Mechanisms of Memory Consolidation That Operate During Sleep. Front Mol Neurosci. 2021;14:767384. doi: 10.3389/fnmol.2021.767384 34867190 PMC8636908

[19] Loprinzi PD, Roig M, Etnier JL, Tomporowski PD, Voss M. Acute and Chronic Exercise Effects on Human Memory: What We Know and Where to Go from Here. J Clin Med. 2021;10(21):4812. doi: 10.3390/jcm10214812 34768329 PMC8584999

[20] Marin Bosch B, Bringard A, Logrieco MG, Lauer E, Imobersteg N, Thomas A, et al. A single session of moderate intensity exercise influences memory, endocannabinoids and brain derived neurotrophic factor levels in men. Sci Rep. 2021;11(1):14371. doi: 10.1038/s41598-021-93813-5 34257382 PMC8277796

[21] Sui W, Smith ST, Fagan MJ, Rollo S, Prapavessis H. The effects of sedentary behaviour interventions on work-related productivity and performance outcomes in real and simulated office work: A systematic review. Appl Ergon. 2019;75:27–73. doi: 10.1016/j.apergo.2018.09.002 30509536

[22] Dupont F, Léger P-M, Begon M, Lecot F, Sénécal S, Labonté-Lemoyne E, et al. Health and productivity at work: which active workstation for which benefits: a systematic review. Occup Environ Med. 2019;76(5):281–94. doi: 10.1136/oemed-2018-105397 30692162

[23] Zhou L, Deng X, Xu M, Wu Y, Shang X, E F, et al. The effects of active workstations on reducing work-specific sedentary time in office workers: a network meta-analysis of 23 randomized controlled trials. Int J Behav Nutr Phys Act. 2023;20(1):92. doi: 10.1186/s12966-023-01467-5 37501138 PMC10375647

[24] Polo-Recuero B, Rojo-Tirado MÁ, Ordóñez-Dios A, Breitkreuz D, Lorenzo A. The Effects of Bike Desks in Formal Education Classroom-Based Physical Activity: A Systematic Review. Sustainability. 2021;13(13):7326. doi: 10.3390/su13137326

[25] Grosprêtre S, Ennequin G, Peseux S, Isacco L. Feasibility and acceptability of “active” classroom workstations among French university students and lecturers: a pilot study. BMC Public Health. 2021;21(1):1001. doi: 10.1186/s12889-021-11074-3 34044813 PMC8161641

[26] Hoppenfeld J, Graves SJ, Sewell RR, Halling TD. Biking to Academic Success: A Study on a Bike Desk Implementation at an Academic Library. Public Services Quarterly. 2019;15(2):85–103. doi: 10.1080/15228959.2018.1552229

[27] Bastien Tardif C, Cantin M, Sénécal S, Léger P-M, Labonté-Lemoyne É, Begon M, et al. Implementation of Active Workstations in University Libraries-A Comparison of Portable Pedal Exercise Machines and Standing Desks. Int J Environ Res Public Health. 2018;15(6):1242. doi: 10.3390/ijerph15061242 29895760 PMC6024930

[28] Maeda H, Quartiroli A, Vos PW, Carr LJ, Mahar MT. Feasibility of retrofitting a university library with active workstations to reduce sedentary behavior. Am J Prev Med. 2014;46(5):525–8. doi: 10.1016/j.amepre.2014.01.024 24745643

[29] Pilcher JJ, Morris DM, Bryant SA, Merritt PA, Feigl HB. Decreasing Sedentary Behavior: Effects on Academic Performance, Meta-Cognition, and Sleep. Front Neurosci. 2017;11:219. doi: 10.3389/fnins.2017.00219 28536499 PMC5422426

[30] Labban JD, Etnier JL. Effects of acute exercise on long-term memory. Res Q Exerc Sport. 2011;82(4):712–21. doi: 10.1080/02701367.2011.10599808 22276413

[31] Polar Beat. v: 3.5.6 ed. iOS app store: Polar Electro Oy, Kempele, Finland; 2023.

[32] Borchert K. Verbal Paired Associates Learning Task. Inquisit 6: Millisecond Software, LLC; 2022.

[33] Craig CL, Marshall AL, Sjöström M, Bauman AE, Booth ML, Ainsworth BE, et al. International physical activity questionnaire: 12-country reliability and validity. Med Sci Sports Exerc. 2003;35(8):1381–95. doi: 10.1249/01.MSS.0000078924.61453.FB 12900694

[34] IBM. Statistical Package for the Social Sciences (SPSS). 29 ed: SPSS Inc.; 2023.

[35] Yang YJ. An Overview of Current Physical Activity Recommendations in Primary Care. Korean J Fam Med. 2019;40(3):135–42. doi: 10.4082/kjfm.19.0038 31122003 PMC6536904

[36] Tomporowski PD. Effects of acute bouts of exercise on cognition. Acta Psychol (Amst). 2003;112(3):297–324. doi: 10.1016/s0001-6918(02)00134-8 12595152

[37] Dunbar CC, Robertson RJ, Baun R, Blandin MF, Metz K, Burdett R, et al. The validity of regulating exercise intensity by ratings of perceived exertion. Med Sci Sports Exerc. 1992;24(1):94–9. doi: 10.1249/00005768-199201000-00016 1549002

[38] Nanda B, Balde J, Manjunatha S. The Acute Effects of a Single Bout of Moderate-intensity Aerobic Exercise on Cognitive Functions in Healthy Adult Males. J Clin Diagn Res. 2013;7(9):1883–5. doi: 10.7860/JCDR/2013/5855.3341 24179888 PMC3809627

[39] Loprinzi PD, Kane CJ. Exercise and cognitive function: a randomized controlled trial examining acute exercise and free-living physical activity and sedentary effects. Mayo Clin Proc. 2015;90(4):450–60. doi: 10.1016/j.mayocp.2014.12.023 25746399

[40] van Dongen EV, Kersten IHP, Wagner IC, Morris RGM, Fernández G. Physical Exercise Performed Four Hours after Learning Improves Memory Retention and Increases Hippocampal Pattern Similarity during Retrieval. Curr Biol. 2016;26(13):1722–7. doi: 10.1016/j.cub.2016.04.071 27321998

[41] McNerney MW, Radvansky GA. Mind racing: The influence of exercise on long-term memory consolidation. Memory. 2015;23(8):1140–51. doi: 10.1080/09658211.2014.962545 25312348

[42] Bantoft C, Summers MJ, Tranent PJ, Palmer MA, Cooley PD, Pedersen SJ. Effect of Standing or Walking at a Workstation on Cognitive Function: A Randomized Counterbalanced Trial. Hum Factors. 2016;58(1):140–9. doi: 10.1177/0018720815605446 26408647

[43] Ruiter M, Görlich E, Loyens S, Wong J, Paas F. Effects of Desk-Bike Cycling on Phonological Working Memory Performance in Adolescents With Attention Deficit Hyperactivity Disorder. Front Educ. 2022;7. doi: 10.3389/feduc.2022.841576

[44] Wamsley EJ. Offline memory consolidation during waking rest. Nat Rev Psychol. 2022;1(8):441–53. doi: 10.1038/s44159-022-00072-w

[45] Torbeyns T, de Geus B, Bailey S, De Pauw K, Decroix L, Van Cutsem J, et al. Cycling on a Bike Desk Positively Influences Cognitive Performance. PLoS One. 2016;11(11):e0165510. doi: 10.1371/journal.pone.0165510 27806079 PMC5091773

[46] Hervieux V, Tremblay A, Biron C. Active meetings on stationary bicycle: An intervention to promote health at work without impairing performance. Appl Ergon. 2021;90:103269. doi: 10.1016/j.apergo.2020.103269 32956981

[47] Makepeace R, Craig M. Higher intensity exercise after encoding is more conducive to episodic memory retention than lower intensity exercise: A field study in endurance runners. PLoS One. 2024;19(9):e0308373. doi: 10.1371/journal.pone.0308373 39269940 PMC11398685

[48] Rodríguez-Gutiérrez E, Torres-Costoso A, Saz-Lara A, Bizzozero-Peroni B, Guzmán-Pavón M, Sánchez-López M, et al. Effectiveness of high-intensity interval training on peripheral brain-derived neurotrophic factor in adults: A systematic review and network meta-analysis. Scandinavian Journal of Medicine & Science in Sports. 2024;34(1). 10.1111/sms.14496. PubMed PMID: WOS:001068191800001. 37728896

[49] Luft CDB, Takase E, Darby D. Heart rate variability and cognitive function: effects of physical effort. Biol Psychol. 2009;82(2):164–8. doi: 10.1016/j.biopsycho.2009.07.007 19632295

[50] Benzing V, Heinks T, Eggenberger N, Schmidt M. Acute Cognitively Engaging Exergame-Based Physical Activity Enhances Executive Functions in Adolescents. PLoS One. 2016;11(12):e0167501. doi: 10.1371/journal.pone.0167501 28030542 PMC5193332

[51] Loprinzi PD, Wade B. Exercise and cardiorespiratory fitness on subjective memory complaints. Psychol Health Med. 2019;24(6):749–56. doi: 10.1080/13548506.2018.1557713 30526017

[52] Rigdon B, Loprinzi PD. The Association of Cardiorespiratory Fitness on Memory Function: Systematic Review. Medicina (Kaunas). 2019;55(5):127. doi: 10.3390/medicina55050127 31075908 PMC6572478

[53] Mou H, Fang Q, Tian S, Qiu F. Effects of acute exercise with different modalities on working memory in men with high and low aerobic fitness. Physiol Behav. 2023;258:114012. doi: 10.1016/j.physbeh.2022.114012 36341835

[54] Laurent JS, Watts R, Adise S, Allgaier N, Chaarani B, Garavan H, et al. Associations Among Body Mass Index, Cortical Thickness, and Executive Function in Children. JAMA Pediatr. 2020;174(2):170–7. doi: 10.1001/jamapediatrics.2019.4708 31816020 PMC6902097

[55] Illam S, Lee GJ, Rajaram S, Sabate J. 13 The Relationship Between Body Mass Index (BMI) and Cognitive Performance Among Overweight Adults. J Int Neuropsychol Soc. 2023;29(s1):531–531. doi: 10.1017/s1355617723006847

[56] Erickson KI, Leckie RL, Weinstein AM. Physical activity, fitness, and gray matter volume. Neurobiol Aging. 2014;35 Suppl 2:S20–8. doi: 10.1016/j.neurobiolaging.2014.03.034 24952993 PMC4094356

[57] Sun X, Li Y, Cai L, Wang Y. Effects of physical activity interventions on cognitive performance of overweight or obese children and adolescents: a systematic review and meta-analysis. Pediatr Res. 2021;89(1):46–53. doi: 10.1038/s41390-020-0941-3 32388536

[58] Potkin KT, Bunney WE Jr. Sleep improves memory: the effect of sleep on long term memory in early adolescence. PLoS One. 2012;7(8):e42191. doi: 10.1371/journal.pone.0042191 22879917 PMC3413705

[59] Puri S, Shaheen M, Grover B. Nutrition and cognitive health: A life course approach. Front Public Health. 2023;11:1023907. doi: 10.3389/fpubh.2023.1023907 37050953 PMC10083484

[60] Raise-Abdullahi P, Raeis-Abdollahi E, Meamar M, Rashidy-Pour A. Effects of coffee on cognitive function. Prog Brain Res. 2024;288:133–66. doi: 10.1016/bs.pbr.2024.06.016 39168555

