# Supplementary material for: The impact of acute bike desk usage before encoding and during early consolidation on memory task performance in university students and use case evaluation in an educational setting
Source: PLoS One. 2025 Mar 17;20(3):e0319658. doi: 10.1371/journal.pone.0319658 (PMC11913288; doi:10.1371/journal.pone.0319658)
Supplement: S1 File — (DOCX) [file pone.0319658.s001.docx]

**Sample size calculation**

$$n= \frac{{2\left( Z_{\alpha}+Z_{\beta} \right)}^{2}}{d^{2}}$$

$$n= \frac{{2\left( 1.96+0.84 \right)}^{2}}{{0.8}^{2}}$$

$$n=25$$

n = Required sample size

d = Effect size (value = 0.8 based on prior literature)

α = Significance (value = 0.05)

β = Power (value = 80%)

*Z*_α_ = Z-score for α (1.96 for 0.05)

Z_β_ = Z-score for 1 - β (0.84 for β = 80%)
