# Supplementary material for: The impact of acute bike desk usage before encoding and during early consolidation on memory task performance in university students and use case evaluation in an educational setting
Source: PLoS One. 2025 Mar 17;20(3):e0319658. doi: 10.1371/journal.pone.0319658 (PMC11913288; doi:10.1371/journal.pone.0319658)
Supplement: S2 Appendix — (DOCX) [file pone.0319658.s002.docx]

**S2 Apprendix.**

**Table 1 Summary data of participants’ responses to evaluation questionnaire, part 1.**

|  | **Strongly agree** | **Agree** | **Neutral** | **Disagree** | **Strongly disagree** |
| --- | --- | --- | --- | --- | --- |
| I found the bike desk seat comfortable to sit on. | 3 (11.5) | 3 (11.5) | 2 (7.7) | 8 (30.8) | 10 (38.5) |
| I found the bike desk height comfortable while cycling. | 8 (30.8) | 12 (46.2) | 1 (3.8) | 2 (7.7) | 3 (11.5) |
| I was able to concentrate on the prescribed videos without distraction while using the bike desk. | 8 (30.8) | 4 (15.4) | 8 (30.8) | 5 (19.2) | 1 (3.8) |
| I could sustain the 'fairly light' to 'somewhat hard' exertion levels during cycling. | 14 (53.8) | 10 (38.5) | 1 (3.8) | 1 (3.8) | 0 (0.0) |
| I could sustain 'fairly light' to 'somewhat hard' exertion levels while engaging in academic-related tasks. | 5 (19.2) | 15 (57.7) | 3 (11.5) | 2 (7.7) | 1 (3.8) |
| I think bike desks have the potential to reduce my sedentary time without sacrificing my study time. | 3 (11.5) | 13 (50.0) | 4 (15.4) | 5 (19.2) | 1 (3.8) |
| I found the bike desk fun to use. | 11 (42.3) | 8 (30.8) | 6 (23.1) | 1 (3.8) | 0 (0.0) |
| I found that the cycling distracted me from focusing on task. | 1 (3.8) | 10 (38.5) | 4 (15.4) | 8 (30.8) | 3 (11.5) |
| I would attend the library more often to use the bike desks. | 3 (11.5) | 9 (34.6) | 6 (23.1) | 6 (23.1) | 2 (7.7) |
| I would engage in academic tasks on a bike desk in the library. | 3 (11.5) | 10 (38.5) | 7 (26.9) | 4 (15.4) | 2 (7.7) |
| I would use a bike desk in the library for a break from academic tasks. | 6 (23.1) | 11 (42.3) | 3 (11.5) | 4 (15.4) | 2 (7.7) |
| I would recommend my friends and colleagues to use a bike desk in the library. | 8 (30.8) | 10 (38.5) | 7 (26.9) | 0 (0.0) | 1 (3.8) |
| I would use a bike desk in plain view of other students in the library. | 2 (7.7) | 2 (7.7) | 6 (23.1) | 7 (26.9) | 9 (34.6) |
| I would use a bike desk alone in a private space in the library. | 7 (26.9) | 14 (53.8) | 0 (0.0) | 3 (11.5) | 2 (7.7) |
| I would use a bike desk with other students in a group space in the library. | 4 (15.4) | 5 (19.2) | 6 (23.1) | 7 (26.9) | 4 (15.4) |
| I would use a bike desk with other students in an active study gym in the sports hub | 7 (26.9) | 7 (26.9) | 5 (19.2) | 4 (15.4) | 3 (11.5) |
|  |  |  |  |  |  |
| Indicate how often you would use a bike desk in the following locations: | **Daily** | **2-3 times a week** | **Once a week** | **Once a month** | **Never** |
| Open, private, or group space in the library | 1 (3.8) | 11 (42.3) | 8 (30.8) | 3 (11.5) | 3 (11.5) |
| Active study gym in the sports hub | 2 (7.7) | 9 (34.6) | 4 (15.4) | 5 (19.2) | 6 (23.1) |
|  |  |  |  |  |  |
|  | **Watching educational videos** | **Listening to educational podcasts** | **Reading course material** | **Making notes** | **Doing flash cards** |
| What sort of academic activity are you likely to engage in while cycling at a bike desk? | 20 (76.9) | 17 (65.4) | 9 (34.6) | 6 (23.1) | 1 (7.7) |

**Table 2 Summary data of participants’ responses to evaluation questionnaire, part 2.**

|  | **Yes** | **No** |
| --- | --- | --- |
| Do you think using a bike desk would improve your study experience? | 18 (69.2) | 8 (30.8) |
|  |  |  |
|  | **Cycling** | **Seated** |
| In which session were you more focused on the educational videos? | 12 (46.2) | 14 (53.8) |
| In which session do you think you performed better in the computer-based memory test? | 14 (53.8) | 12 (46.2) |
